# Secondary Hemochromatosis due to Chronic Oral Iron Supplementation

**DOI:** 10.1155/2017/2494167

**Published:** 2017-01-04

**Authors:** Ronald Lands, Emmanuel Isang

**Affiliations:** The University of Tennessee, Graduate School of Medicine, Knoxville, TN, USA

## Abstract

Iron may accumulate in excess due to a mutation in the HFE gene that upregulates absorption or when it is ingested or infused at levels that exceed the body's ability to clear it. Excess iron deposition in parenchymal tissue causes injury and ultimately organ dysfunction. Diabetes mellitus and hepatic cirrhosis due to pancreas and liver damage are just two examples of diseases that result from iron overload. Despite the rapid growth of information regarding iron metabolism and iron overload states, the most effective treatment is still serial phlebotomies. We present a patient who developed iron overload due to chronic ingestion of oral ferrous sulfate. This case illustrates the importance of querying geriatric patients regarding their use of nonprescription iron products without a medical indication.

## 1. Introduction

Iron overload syndromes may be genetic or acquired. Hemochromatosis, a common genetic disorder in Caucasians, is the result of a mutation in the HFE gene that causes iron to be absorbed in excess. Iron overload may also accompany anemias characterized by significant ineffective erythropoiesis, chronic transfusions, or both as is the case in thalassemia. There is no excretory organ for iron, so the only mechanism for its clearance is through epithelial sloughing or bleeding. A sustained imbalance of iron intake and absorption that is greater than the ability to remove it results in iron overload. We describe a patient who was supplemented with oral ferrous sulfate, 1–3 325 mg tablets per day, for 30 years after menopause because of a mistaken belief that it would provide some benefit in her general health. She developed iron overload despite the absence of any of the common detectable HFE mutations.

## 2. Case Report

A 78-year-old Caucasian female was referred to hematology after her primary physician noted a persistent elevation in her serum ferritin level. Her primary concern was a profound sense of fatigue and recent erratic control of her thyroid medication despite years of stability. She had developed atrial fibrillation within the year prior to detection of the elevated ferritin. In the remote past, she had breast cancer treated with lumpectomy, radiation therapy, and adjuvant chemotherapy. She was actively being treated for hyperlipidemia, gastroesophageal reflux with Barrett's esophagus, and vitamin B12 deficiency. There was no family history of unexplained liver disease.

She was a healthy appearing woman who looked younger than her stated age. Her sclera were not icteric. The liver and spleen were not palpable. She had no stigmata of chronic liver disease. Her cardiac rhythm was regular with a normal rate.

Laboratory data revealed hemoglobin of 12.5 g/dL (12.0–16.0) with normal WBC and platelets. Liver transaminases and alkaline phosphatase levels were normal. PT was 10.7 sec (9.1–12.0), and INR was 0.97 (0.90–1.10). Serum ferritin was 1,379 ng/mL (10–162 ng/mL). Serum iron was 77 *μ*g/dL (25–156), TIBC was 226 *μ*g/dL (250–450), and transferrin saturation was 34% (20–50). She did not carry any of the mutations C282Y, H63D, or S65C.

**Figure 1:**
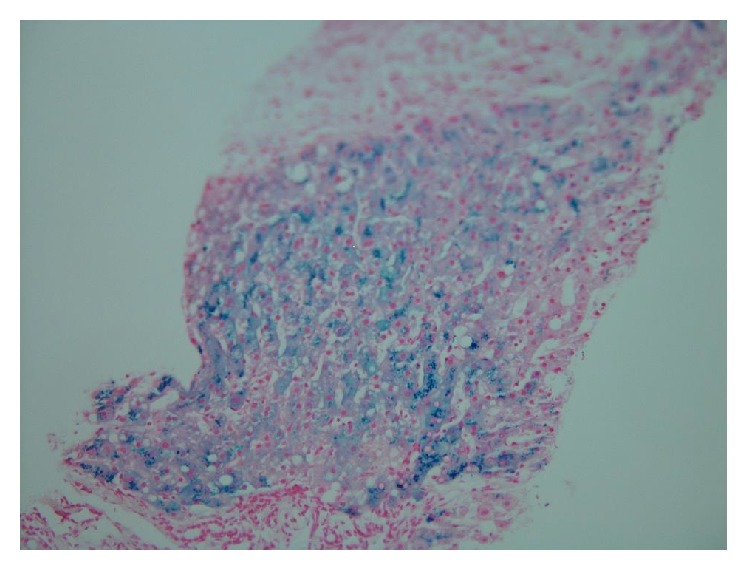
Liver biopsy demonstrating increased iron content in hepatocytes and Kupffer cells.

MRI of the abdomen demonstrated iron deposition in the liver and spleen compatible with secondary hemochromatosis. Liver biopsy documented increased iron uptake in the hepatocytes and Kupffer cells (Figure 1). There was focal periportal fibrosis but no cirrhosis. The dry weight iron content in a biopsy of liver tissue was 6,153 *μ*g/g (270–1,600 *μ*g/g).

The iron supplements were discontinued. She was prescribed one 300–350 mL phlebotomy of whole blood per week. After approximately 4 phlebotomies, her hemoglobin fell to 9.7 while her ferritin remained elevated. Erythropoietin injections were added in an attempt to minimize delays and interruptions in the phlebotomy schedule, and with some difficulty, she tolerated 17 phlebotomies over a span of 5 months. Her hemoglobin the week following her 17th phlebotomy was 8 g/dL despite the erythropoietin stimulants. Her ferritin at that point was 65 ng/mL. She had one more phlebotomy about six months later when her ferritin rose again to more than 100 ng/mL. After that, because of the symptoms induced by the procedure and based on the rationale that she had no apparent mutation causing excessive absorption, we have accepted the normal range for our lab as our goal. To date, the iron saturation has remained less than 30% and her ferritin has remained stable between 70 and 100 ng/mL.

## 3. Discussion

Iron is a micronutrient which if deficient or excessive may cause morbidity and mortality. In 1889, Von Reclinghasen observed the association of iron accumulation in the pancreas and the development of diabetes due to its associated tissue injury. Knowledge about hemochromatosis has grown dramatically from the era when Von Reclinghasen made his observations to the current understanding of iron homeostasis [1, 2].

The availability of molecular genotyping of the HFE gene highlights the paradox of a common mutation but a rare disease. The prevalence of detectable mutations in North America is between 1 in 200 and 1 in 500 and is even more common in some northern European countries. They are transmitted, with rare exceptions, in an autosomal recessive pattern. Most people with mutations never develop the disease. Likewise, a significant number of patients with the clinical phenotype of primary hemochromatosis have no detectable HFE mutation [3].

The liver is the conductor of systemic iron balance, sensing a variety of iron related signals and modulating iron absorption and storage by hepcidin expression [4]. The relationship of serum ferritin and total body iron stores has been clearly established. As the ferritin increases, the risk of significant liver disease rises [5]. It may be elevated in the absence of iron overload, however, and competing comorbidities such as alcoholic liver disease, hepatic steatosis, or viral infections may confuse the diagnosis because of the clinical similarities.

The diagnosis of iron overload is often overlooked because the signs and symptoms are commonly associated with other diseases, such as chronic fatigue, cirrhosis, diabetes, congestive heart failure, hypogonadism, osteoporosis, and arthritis. In the absence of acute or chronic inflammation, screening for iron overload is warranted upon discovery of ferritin levels above 200 ng/mL and transferrin saturation above 45% in women or ferritin more than 300 ng/mL and transferrin saturation greater than 50% in men. Specialized MRI scanning of the liver provides indirect evidence of iron overload. Liver biopsy provides a direct measurement of liver iron concentration along with the pathologist's assessment of liver histology [6].

A wide array of treatment strategies exploiting the role of hepcidin in the regulation of iron homeostasis are under development [7]. Currently, however, the treatment of hemochromatosis whether genetic or acquired is serial phlebotomies continuing until the ferritin is less than 50 ng/mL and iron saturation is less than 50%. Those patients with genetic hyperabsorption of iron will require periodic phlebotomies indefinitely to maintain their iron stores at a safe level. As in our patient, iron overload due to chronic ingestion of iron in the absence of mutations that upregulate iron absorption should not require phlebotomies after reaching acceptable levels of ferritin and iron saturation.

## 4. Conclusion

Nutritional iron overload requires years of supplementation to develop. Patients may take over the counterpreparations of iron supplements for a perceived health benefit and be unaware that it carries potential risks when taken for a long period of time. Medical professionals of all specialties should query their patients as to whether they take supplements with iron and discontinue it if there is no medical indication.

## References

[1] Von Reclinghasen F. D. (1889). *Hämochromatose*.

[2] Andrews N. C. (2008). Forging a field: the golden age of iron biology. *Blood*.

[3] Thachil J., Solberg L. A., Kahn M. J., Mccrae K. R., Mccrae K., Steensma D. (2013). Iron metabolism, iron overload, and the porphyrias. *American Society of Hematology—Self Assessment Program*.

[4] Meynard D., Babitt J. L., Lin H. Y. (2014). The liver: conductor of systemic iron balance. *Blood*.

[5] Adams P. C. (2015). Epidemiology and diagnostic testing for hemochromatosis and iron overload. *International Journal of Laboratory Hematology*.

[6] Fleming R. E., Ponka P. (2012). Iron overload in human disease. *New England Journal of Medicine*.

[7] Arezes J., Nemeth E. (2015). Hepcidin and iron disorders: new biology and clinical approaches. *International Journal of Laboratory Hematology*.

